# Dicarbon­yl(η^5^-cyclo­penta­dien­yl)(hexa­methyl­enetetra­mine-κ*N* ^1^)iron(II) tetra­fluoridoborate

**DOI:** 10.1107/S1600536812031649

**Published:** 2012-07-18

**Authors:** Cyprian M. M’thiruaine, Holger B. Friedrich, Bernard Omondi

**Affiliations:** a School of Chemistry, University of KwaZulu-Natal, Private Bag X54001, Durban 4000, South Africa

## Abstract

In the structure of the title compound, [Fe(C_5_H_5_)(C_6_H_12_N_4_)(CO)_2_]BF_4_, the arrangement around the Fe^II^ atom corresponds to that of a three-legged piano stool. The cyclo­penta­dienyl ligand occupies three coordination sites of the apical position in a η^5^ fashion, while two CO ligands and one N atom of the hexa­methyl­ene­tetra­mine ligand occupy the remaining coordination sites to complete a distorted octa­hedral geometry. The asymmetric unit consists of two sets of crystallographically independent cations and anions with the r.m.s. deviations of the overlay of non-H atoms of each pair being 0.081 and 0.120 Å, respectively. The Fe—N bond lengths are 2.0459 (15) and 2.0490 (14) Å, while the Fe—Cp(centroid) distances are 1.7257 (3) and 1.7246 (3) Å. One of the anions displays disorder, with the F atoms having occupancies of 0.58 (4) and 0.42 (4).

## Related literature

For the synthesis of the title compound and structure of the dinuclear compound [Fe_2_(η^5^-C_5_H_5_)_2_(C_6_H_12_N_4_)(CO)_4_](BF_4_)_2_, see: M’thiruaine, Friedrich, Changamu & Bala (2012 ▶). For other related compounds, see: Matos & Verkade (2003 ▶); M’thiruaine, Friedrich, Changamu & Fernandes (2012 ▶); M’thiruaine *et al.* (2011 ▶).
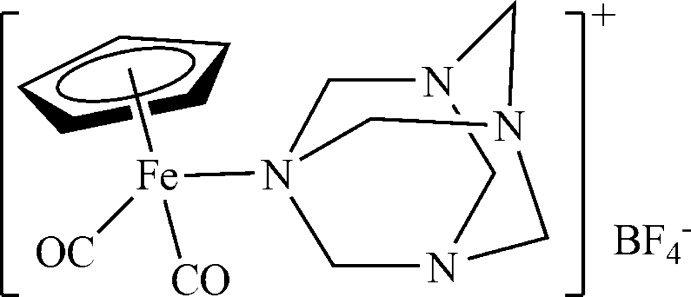


## Experimental

### 

#### Crystal data

[Fe(C_5_H_5_)(C_6_H_12_N_4_)(CO)_2_]BF_4_
*M* *_r_* = 403.97
Monoclinic, 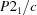

*a* = 15.1054 (6) Å
*b* = 14.6407 (6) Å
*c* = 14.2267 (6) Å
β = 96.997 (2)°
*V* = 3122.9 (2) Å^3^
*Z* = 8
Mo *K*α radiation
μ = 1.03 mm^−1^
*T* = 173 K
0.42 × 0.19 × 0.16 mm

#### Data collection

Bruker SMART APEXII CCD diffractometer
Absorption correction: multi-scan (*SADABS*; Bruker, 2008 ▶) *T*_min_ = 0.673, *T*_max_ = 0.853
62504 measured reflections
7768 independent reflections
6765 reflections with *I* > 2σ(*I*)
*R*_int_ = 0.033

#### Refinement

*R*[*F* ^2^ > 2σ(*F* ^2^)] = 0.034
*wR*(*F* ^2^) = 0.092
*S* = 1.08
7768 reflections
488 parameters
12 restraints
H-atom parameters constrained
Δρ_max_ = 0.63 e Å^−3^
Δρ_min_ = −0.43 e Å^−3^

### 

Data collection: *APEX2* (Bruker, 2008 ▶); cell refinement: *SAINT-Plus* (Bruker, 2008 ▶); data reduction: *SAINT-Plus* and *XPREP* (Bruker, 2008 ▶); program(s) used to solve structure: *SHELXS97* (Sheldrick, 2008 ▶); program(s) used to refine structure: *SHELXL97* (Sheldrick, 2008 ▶); molecular graphics: *ORTEP-3* (Farrugia, 1997 ▶); software used to prepare material for publication: *WinGX* (Farrugia, 1999 ▶).

## Supplementary Material

Crystal structure: contains datablock(s) global, I. DOI: 10.1107/S1600536812031649/kj2206sup1.cif

Structure factors: contains datablock(s) I. DOI: 10.1107/S1600536812031649/kj2206Isup2.hkl

Additional supplementary materials:  crystallographic information; 3D view; checkCIF report

## Figures and Tables

**Fig. 1.**
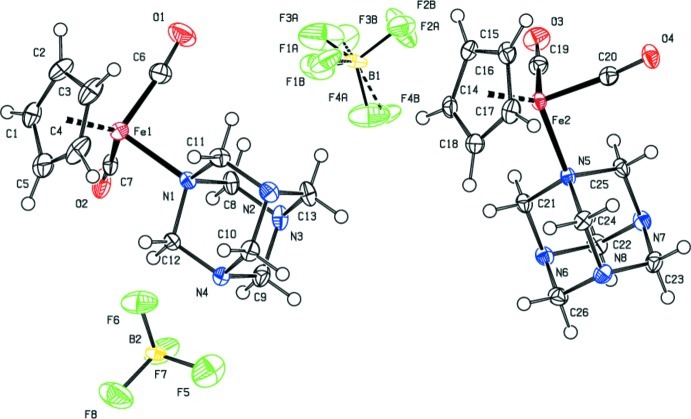
Fig. 1: Molecular structure of the title complex with the atom labeling scheme. Ellipsoids are drawn at 50% probability level.

## supplementary crystallographic information

### Comment

The title compound was obtained as part of our ongoing investigation of the reactions of substitutionally unsaturated metal complexes with nitrogen donor ligands. The synthesis and characterization data was previously reported by us. (M'thiruaine *et al.*, 2011; M'thiruaine, Friedrich, Changamu & Fernandes, 2012). The asymmetric unit consists of two independent molecules of [Fe(η^5^-C_5_H_5_){N_4_(CH_2_)_6_}(CO)_2_]^+^ and two molecules of BF_4_^-^. The structure exhibits a typical three legged piano stool structure with Fe^II^ coordinated by one nitrogen atom of the hexamethylenetetramine ligand and two of the CO ligands. The coordination geometry around Fe is that of the distorted octahedral in which three sites are occupied by the η^5^-cyclopentadienyl ligand, two by the CO ligands and one by an N atom of the hexamethylenetetramine. Its structure is closely similar to that of recently reported pentamethylcyclopentadienyl analogue compound [Fe{η^5^-C_5_(CH_3_)_5_}{N_4_(CH_2_)_6_}(CO)_2_]BF_4_ (M'thiruaine, Friedrich, Changamu & Fernandes, 2012). One of the anions displays disorder with the fluorine atoms having occupancies of 0.58 (4) for F1A, F2A, F3A and F4A, and 0.42 (4) for F1B, F2B, F3B and F4B.

The asymmetric unit consist of two sets of crystallographically independent molecules of the cations [r.m.s = 0.081 Å] and anions [r.m.s. = 0.120 Å]. The cations are related by a non-crystallographic translation across the *ac* diagonal. The Fe—N bonds distances are 2.0458 (15) and 2.0489 (14) Å, which are slightly shorter than the correspomding distances of the Cp* analogue (M'thiruaine, Friedrich, Changamu & Fernandes, 2012) (2.069 (2) Å) and of the dinuclear complex [{Cp(CO)_2_Fe}_2_{N_4_(CH_2_)_6_}]^2+^ (M'thiruaine, Friedrich, Changamu & Bala, 2012) (2.0817 (17) and 2.0858 (18) Å) as well as that of [(CO)_4_Fe{N_2_(CH_2_)_6_}] (Matos and Verkade, 2003) (2.092 (4) Å).

### Experimental

The title compound was prepared according to a reported procedure (M'thiruaine, Friedrich, Changamu & Bala, 2012) and crystals were grown by layering a concentrated solution of the compound in acetone with Et_2_O and the mixture kept undisturbed in the dark for four weeks.

### Refinement

Carbon-bound H-atoms were placed in calculated positions [C—H = 0.97 Å for for methylene H atoms and 0.98 for methine H atoms; *U*_iso_(H) = 1.2*U*_eq_(C)] and were included in the refinement in the riding model approximation. One of the tetrafluoroborate anions was refined with a disorder model. The total occupancy of each atom was constrained to 1; the occupancy for sites F1A, F2A, F3A and F4A was 0.58 (4) and 0.42 (4) for F1B, F2B, F3B and F4B. F4A and F4B were refined with restraints to prevent extreme anisotropy. One reflection (1 0 0) was omited from the *hkl* file.

### Crystal data

**Table:**

[Fe(C_5_H_5_)(C_6_H_12_N_4_)(CO)_2_]BF_4_	*F*(000) = 1648
*M**_r_* = 403.97	*D*_x_ = 1.718 Mg m^−^^3^
Monoclinic, *P*2_1_/*c*	Mo *K*α radiation, λ = 0.71073 Å
Hall symbol: -P 2ybc	Cell parameters from 63978 reflections
*a* = 15.1054 (6) Å	θ = 1.9–28.3°
*b* = 14.6407 (6) Å	µ = 1.03 mm^−^^1^
*c* = 14.2267 (6) Å	*T* = 173 K
β = 96.997 (2)°	Block, yellow
*V* = 3122.9 (2) Å^3^	0.42 × 0.19 × 0.16 mm
*Z* = 8	

### Data collection

**Table:**

Bruker SMART APEXII CCD diffractometer	6765 reflections with *I* > 2σ(*I*)
Graphite monochromator	*R*_int_ = 0.033
φ and ω scans	θ_max_ = 28.3°, θ_min_ = 1.9°
Absorption correction: multi-scan (*SADABS*; Bruker, 2008)	*h* = −20→20
*T*_min_ = 0.673, *T*_max_ = 0.853	*k* = −19→19
62504 measured reflections	*l* = −18→18
7768 independent reflections	

### Refinement

**Table:**

Refinement on *F*^2^	Primary atom site location: structure-invariant direct methods
Least-squares matrix: full	Secondary atom site location: difference Fourier map
*R*[*F*^2^ > 2σ(*F*^2^)] = 0.034	Hydrogen site location: inferred from neighbouring sites
*wR*(*F*^2^) = 0.092	H-atom parameters constrained
*S* = 1.08	*w* = 1/[σ^2^(*F*_o_^2^) + (0.0391*P*)^2^ + 2.9672*P*] where *P* = (*F*_o_^2^ + 2*F*_c_^2^)/3
7768 reflections	(Δ/σ)_max_ = 0.026
488 parameters	Δρ_max_ = 0.63 e Å^−^^3^
12 restraints	Δρ_min_ = −0.43 e Å^−^^3^

### Special details

**Table:**

Geometry. All e.s.d.'s (except the e.s.d. in the dihedral angle between two l.s. planes) are estimated using the full covariance matrix. The cell e.s.d.'s are taken into account individually in the estimation of e.s.d.'s in distances, angles and torsion angles; correlations between e.s.d.'s in cell parameters are only used when they are defined by crystal symmetry. An approximate (isotropic) treatment of cell e.s.d.'s is used for estimating e.s.d.'s involving l.s. planes.
Refinement. Refinement of *F*^2^ against ALL reflections. The weighted *R*-factor *wR* and goodness of fit *S* are based on *F*^2^, conventional *R*-factors *R* are based on *F*, with *F* set to zero for negative *F*^2^. The threshold expression of *F*^2^ > σ(*F*^2^) is used only for calculating *R*-factors(gt) *etc*. and is not relevant to the choice of reflections for refinement. *R*-factors based on *F*^2^ are statistically about twice as large as those based on *F*, and *R*- factors based on ALL data will be even larger.

### Fractional atomic coordinates and isotropic or equivalent isotropic displacement parameters (Å^2^)

**Table:**

	*x*	*y*	*z*	*U*_iso_*/*U*_eq_	Occ. (<1)
C1	0.86930 (13)	−0.03952 (15)	0.80821 (16)	0.0275 (4)	
H1	0.8674	−0.1007	0.8393	0.033*	
C2	0.87149 (14)	0.04629 (15)	0.85391 (15)	0.0292 (4)	
H2	0.8723	0.0563	0.9235	0.035*	
C3	0.87061 (14)	0.11633 (15)	0.78406 (17)	0.0323 (5)	
H3	0.8709	0.1837	0.7957	0.039*	
C4	0.86568 (13)	0.07273 (17)	0.69515 (16)	0.0339 (5)	
H4	0.8639	0.1044	0.6327	0.041*	
C5	0.86628 (13)	−0.02251 (17)	0.70865 (16)	0.0320 (5)	
H5	0.863	−0.0699	0.6577	0.038*	
C6	1.04710 (15)	0.07326 (13)	0.88830 (14)	0.0270 (4)	
C7	1.04138 (12)	−0.07593 (12)	0.78590 (13)	0.0204 (4)	
C8	1.16153 (11)	0.07250 (12)	0.72516 (14)	0.0199 (4)	
H8A	1.1768	0.0981	0.7895	0.024*	
H8B	1.1723	0.0058	0.7289	0.024*	
C9	1.19672 (12)	0.07558 (13)	0.56646 (14)	0.0235 (4)	
H9A	1.2072	0.0088	0.5678	0.028*	
H9B	1.2362	0.1032	0.5235	0.028*	
C10	1.08860 (12)	0.19357 (13)	0.52845 (13)	0.0214 (4)	
H10A	1.1265	0.2222	0.4846	0.026*	
H10B	1.0256	0.2068	0.5047	0.026*	
C11	1.05207 (13)	0.19222 (12)	0.68625 (13)	0.0200 (4)	
H11A	0.9892	0.2066	0.6627	0.024*	
H11B	1.0654	0.219	0.7503	0.024*	
C12	1.04575 (12)	0.05226 (12)	0.59314 (12)	0.0189 (3)	
H12A	1.0556	−0.0146	0.5947	0.023*	
H12B	0.9826	0.0634	0.5683	0.023*	
C13	1.20368 (13)	0.21211 (12)	0.65816 (15)	0.0241 (4)	
H13A	1.2431	0.2405	0.6156	0.029*	
H13B	1.219	0.2384	0.7222	0.029*	
C14	0.35418 (12)	0.09213 (13)	0.27619 (14)	0.0213 (4)	
H14	0.3491	0.1598	0.2835	0.026*	
C15	0.35489 (12)	0.02563 (13)	0.34992 (14)	0.0219 (4)	
H15	0.3497	0.0387	0.418	0.026*	
C16	0.36150 (12)	−0.06169 (13)	0.30960 (14)	0.0226 (4)	
H16	0.3617	−0.1213	0.344	0.027*	
C17	0.36425 (12)	−0.05030 (14)	0.21025 (14)	0.0237 (4)	
H17	0.3679	−0.1003	0.163	0.028*	
C18	0.35832 (12)	0.04445 (14)	0.19039 (14)	0.0224 (4)	
H18	0.3588	0.073	0.1266	0.027*	
C19	0.52488 (12)	0.07220 (13)	0.39393 (13)	0.0210 (4)	
C20	0.53847 (12)	−0.08684 (12)	0.31050 (13)	0.0192 (3)	
C21	0.54333 (12)	0.17549 (11)	0.19492 (13)	0.0170 (3)	
H21A	0.4811	0.1876	0.1672	0.02*	
H21B	0.5522	0.2034	0.2588	0.02*	
C22	0.69695 (12)	0.19948 (12)	0.17685 (14)	0.0212 (4)	
H22A	0.7383	0.2289	0.1371	0.025*	
H22B	0.7076	0.2266	0.241	0.025*	
C23	0.69925 (12)	0.06230 (13)	0.08670 (14)	0.0216 (4)	
H23A	0.7117	−0.0041	0.0894	0.026*	
H23B	0.7405	0.0909	0.0463	0.026*	
C24	0.54675 (12)	0.03453 (12)	0.10393 (12)	0.0177 (3)	
H24A	0.5587	−0.032	0.107	0.021*	
H24B	0.4843	0.0434	0.0751	0.021*	
C25	0.65477 (11)	0.05894 (12)	0.24201 (13)	0.0182 (3)	
H25A	0.6659	0.085	0.3066	0.022*	
H25B	0.6671	−0.0074	0.2467	0.022*	
C26	0.58916 (13)	0.17684 (12)	0.04086 (13)	0.0210 (4)	
H26A	0.6289	0.2065	−0.0004	0.025*	
H26B	0.5268	0.1879	0.0131	0.025*	
B1	0.32781 (15)	0.21448 (14)	0.93390 (14)	0.0203 (4)	
B2	1.18854 (14)	−0.20775 (13)	0.54535 (13)	0.0169 (4)	
N1	1.06343 (9)	0.08967 (10)	0.69277 (10)	0.0151 (3)	
N2	1.11003 (11)	0.23350 (10)	0.62362 (11)	0.0209 (3)	
N3	1.21930 (10)	0.11287 (11)	0.66204 (12)	0.0222 (3)	
N4	1.10294 (10)	0.09386 (11)	0.52956 (11)	0.0201 (3)	
N5	0.55772 (9)	0.07343 (9)	0.20366 (10)	0.0145 (3)	
N6	0.60404 (10)	0.21776 (10)	0.13595 (11)	0.0189 (3)	
N7	0.71550 (10)	0.10091 (10)	0.18284 (11)	0.0197 (3)	
N8	0.60659 (10)	0.07755 (10)	0.04412 (11)	0.0193 (3)	
O1	1.08297 (13)	0.09476 (12)	0.95985 (11)	0.0436 (4)	
O2	1.07359 (9)	−0.14597 (9)	0.79173 (12)	0.0314 (3)	
O3	0.55295 (10)	0.10444 (11)	0.46397 (10)	0.0318 (3)	
O4	0.57492 (9)	−0.15342 (9)	0.32834 (11)	0.0265 (3)	
F1A	0.2452 (6)	0.2217 (16)	0.8959 (7)	0.071 (3)	0.58 (4)
F2A	0.3595 (6)	0.3019 (6)	0.9694 (4)	0.0415 (16)	0.58 (4)
F3A	0.313 (2)	0.1541 (5)	0.9998 (6)	0.073 (4)	0.58 (4)
F4A	0.3667 (12)	0.1825 (12)	0.8619 (12)	0.056 (3)	0.58 (4)
F1B	0.2404 (8)	0.2517 (10)	0.8923 (7)	0.045 (2)	0.42 (4)
F2B	0.3690 (8)	0.2897 (11)	0.9743 (7)	0.065 (3)	0.42 (4)
F3B	0.3532 (9)	0.1541 (7)	1.0064 (8)	0.0482 (19)	0.42 (4)
F4B	0.3880 (12)	0.1945 (13)	0.8637 (15)	0.040 (2)	0.42 (4)
F5	1.23942 (12)	−0.14457 (10)	0.50207 (12)	0.0563 (4)	
F6	1.13743 (11)	−0.16773 (11)	0.60532 (11)	0.0495 (4)	
F7	1.24900 (10)	−0.26959 (10)	0.59476 (9)	0.0434 (4)	
F8	1.13740 (10)	−0.25675 (15)	0.47543 (13)	0.0673 (6)	
Fe1	0.983683 (17)	0.031636 (17)	0.782706 (18)	0.01738 (7)	
Fe2	0.473433 (16)	0.015894 (16)	0.289440 (17)	0.01452 (7)	

### Atomic displacement parameters (Å^2^)

**Table:**

	*U*^11^	*U*^22^	*U*^33^	*U*^12^	*U*^13^	*U*^23^
C1	0.0159 (9)	0.0308 (10)	0.0367 (11)	0.0006 (7)	0.0069 (8)	0.0042 (8)
C2	0.0257 (10)	0.0341 (11)	0.0306 (11)	0.0085 (8)	0.0141 (8)	0.0059 (8)
C3	0.0240 (10)	0.0317 (11)	0.0442 (12)	0.0125 (8)	0.0162 (9)	0.0123 (9)
C4	0.0137 (9)	0.0554 (14)	0.0330 (11)	0.0105 (9)	0.0052 (8)	0.0164 (10)
C5	0.0116 (8)	0.0503 (13)	0.0339 (11)	−0.0003 (8)	0.0016 (8)	−0.0037 (10)
C6	0.0370 (11)	0.0218 (9)	0.0228 (10)	−0.0029 (8)	0.0054 (8)	0.0008 (7)
C7	0.0140 (8)	0.0209 (9)	0.0266 (9)	−0.0040 (7)	0.0033 (7)	0.0007 (7)
C8	0.0122 (8)	0.0188 (8)	0.0273 (9)	−0.0004 (6)	−0.0028 (7)	0.0041 (7)
C9	0.0171 (9)	0.0209 (9)	0.0341 (10)	0.0003 (7)	0.0094 (8)	−0.0011 (7)
C10	0.0193 (9)	0.0230 (9)	0.0215 (9)	0.0006 (7)	0.0009 (7)	0.0033 (7)
C11	0.0255 (9)	0.0126 (8)	0.0221 (9)	0.0045 (7)	0.0036 (7)	0.0003 (6)
C12	0.0169 (8)	0.0205 (8)	0.0194 (8)	−0.0031 (7)	0.0028 (7)	−0.0050 (7)
C13	0.0223 (9)	0.0172 (8)	0.0312 (10)	−0.0060 (7)	−0.0027 (8)	0.0019 (7)
C14	0.0136 (8)	0.0208 (9)	0.0298 (10)	0.0041 (7)	0.0032 (7)	0.0024 (7)
C15	0.0153 (8)	0.0264 (9)	0.0251 (9)	0.0025 (7)	0.0062 (7)	0.0012 (7)
C16	0.0131 (8)	0.0205 (9)	0.0344 (10)	−0.0022 (7)	0.0035 (7)	0.0038 (7)
C17	0.0136 (8)	0.0263 (9)	0.0305 (10)	−0.0030 (7)	0.0001 (7)	−0.0068 (8)
C18	0.0122 (8)	0.0312 (10)	0.0233 (9)	−0.0005 (7)	0.0002 (7)	0.0038 (7)
C19	0.0187 (9)	0.0212 (9)	0.0235 (9)	−0.0013 (7)	0.0037 (7)	0.0003 (7)
C20	0.0156 (8)	0.0183 (8)	0.0241 (9)	−0.0049 (7)	0.0043 (7)	0.0010 (7)
C21	0.0184 (8)	0.0106 (7)	0.0221 (9)	0.0006 (6)	0.0022 (7)	0.0001 (6)
C22	0.0180 (9)	0.0172 (8)	0.0278 (9)	−0.0060 (7)	0.0002 (7)	0.0013 (7)
C23	0.0161 (8)	0.0208 (9)	0.0290 (10)	−0.0001 (7)	0.0064 (7)	0.0004 (7)
C24	0.0170 (8)	0.0172 (8)	0.0193 (8)	−0.0036 (6)	0.0035 (7)	−0.0049 (6)
C25	0.0123 (8)	0.0177 (8)	0.0239 (9)	−0.0005 (6)	−0.0004 (7)	0.0043 (7)
C26	0.0208 (9)	0.0207 (9)	0.0215 (9)	−0.0014 (7)	0.0023 (7)	0.0043 (7)
B1	0.0285 (11)	0.0201 (10)	0.0119 (9)	0.0064 (8)	0.0009 (8)	−0.0050 (7)
B2	0.0206 (10)	0.0182 (9)	0.0112 (8)	0.0048 (7)	−0.0012 (7)	−0.0018 (7)
N1	0.0135 (7)	0.0133 (6)	0.0181 (7)	0.0017 (5)	0.0003 (5)	−0.0015 (5)
N2	0.0240 (8)	0.0148 (7)	0.0235 (8)	0.0009 (6)	0.0010 (6)	0.0012 (6)
N3	0.0146 (7)	0.0182 (7)	0.0331 (9)	−0.0017 (6)	0.0004 (6)	0.0046 (6)
N4	0.0180 (7)	0.0211 (7)	0.0220 (8)	−0.0030 (6)	0.0054 (6)	−0.0028 (6)
N5	0.0130 (7)	0.0109 (6)	0.0191 (7)	−0.0001 (5)	0.0000 (5)	−0.0006 (5)
N6	0.0186 (7)	0.0141 (7)	0.0238 (8)	−0.0017 (5)	0.0018 (6)	0.0025 (6)
N7	0.0138 (7)	0.0178 (7)	0.0274 (8)	−0.0021 (6)	0.0015 (6)	0.0040 (6)
N8	0.0167 (7)	0.0201 (7)	0.0216 (8)	−0.0026 (6)	0.0040 (6)	−0.0012 (6)
O1	0.0653 (12)	0.0411 (9)	0.0227 (8)	−0.0158 (8)	−0.0016 (8)	−0.0032 (7)
O2	0.0222 (7)	0.0177 (7)	0.0560 (10)	0.0011 (5)	0.0115 (7)	0.0059 (6)
O3	0.0325 (8)	0.0376 (8)	0.0243 (7)	−0.0066 (7)	−0.0001 (6)	−0.0066 (6)
O4	0.0189 (7)	0.0173 (6)	0.0435 (8)	0.0020 (5)	0.0049 (6)	0.0076 (6)
F1A	0.035 (2)	0.126 (9)	0.051 (3)	−0.004 (4)	−0.005 (2)	−0.042 (4)
F2A	0.074 (4)	0.0210 (19)	0.035 (2)	−0.0067 (18)	0.028 (3)	−0.0028 (14)
F3A	0.154 (12)	0.0319 (18)	0.038 (2)	0.012 (4)	0.026 (5)	0.0130 (15)
F4A	0.069 (6)	0.059 (5)	0.040 (3)	0.034 (4)	0.010 (4)	−0.005 (3)
F1B	0.039 (3)	0.074 (5)	0.024 (3)	0.009 (3)	0.015 (2)	0.008 (4)
F2B	0.055 (4)	0.049 (5)	0.081 (6)	−0.009 (3)	−0.031 (5)	−0.014 (3)
F3B	0.066 (5)	0.038 (2)	0.042 (3)	0.016 (3)	0.012 (3)	0.022 (2)
F4B	0.050 (5)	0.040 (3)	0.034 (4)	0.021 (3)	0.023 (4)	0.001 (3)
F5	0.0730 (12)	0.0375 (8)	0.0622 (10)	0.0071 (8)	0.0233 (9)	0.0146 (7)
F6	0.0527 (9)	0.0541 (9)	0.0432 (8)	0.0255 (7)	0.0116 (7)	−0.0125 (7)
F7	0.0462 (8)	0.0527 (9)	0.0333 (7)	0.0256 (7)	0.0121 (6)	0.0164 (6)
F8	0.0338 (8)	0.1018 (15)	0.0636 (11)	0.0017 (9)	−0.0049 (7)	−0.0475 (10)
Fe1	0.01647 (13)	0.01728 (13)	0.01851 (13)	0.00332 (9)	0.00265 (10)	0.00090 (9)
Fe2	0.01275 (12)	0.01259 (12)	0.01814 (13)	0.00006 (8)	0.00148 (9)	−0.00002 (9)

### Geometric parameters (Å, º)

**Table:**

C1—C2	1.413 (3)	C16—C17	1.429 (3)
C1—C5	1.433 (3)	C16—Fe2	2.0855 (18)
C1—Fe1	2.087 (2)	C16—H16	1
C1—H1	1	C17—C18	1.416 (3)
C2—C3	1.427 (3)	C17—Fe2	2.1157 (18)
C2—Fe1	2.0887 (19)	C17—H17	1
C2—H2	1	C18—Fe2	2.1418 (18)
C3—C4	1.411 (3)	C18—H18	1
C3—Fe1	2.113 (2)	C19—O3	1.137 (2)
C3—H3	1	C19—Fe2	1.7921 (19)
C4—C5	1.408 (3)	C20—O4	1.133 (2)
C4—Fe1	2.133 (2)	C20—Fe2	1.8016 (18)
C4—H4	1	C21—N6	1.454 (2)
C5—Fe1	2.104 (2)	C21—N5	1.513 (2)
C5—H5	1	C21—H21A	0.99
C6—O1	1.138 (3)	C21—H21B	0.99
C6—Fe1	1.788 (2)	C22—N7	1.471 (2)
C7—O2	1.134 (2)	C22—N6	1.477 (2)
C7—Fe1	1.7978 (19)	C22—H22A	0.99
C8—N3	1.452 (2)	C22—H22B	0.99
C8—N1	1.518 (2)	C23—N7	1.473 (2)
C8—H8A	0.99	C23—N8	1.473 (2)
C8—H8B	0.99	C23—H23A	0.99
C9—N3	1.466 (3)	C23—H23B	0.99
C9—N4	1.474 (2)	C24—N8	1.458 (2)
C9—H9A	0.99	C24—N5	1.519 (2)
C9—H9B	0.99	C24—H24A	0.99
C10—N2	1.474 (2)	C24—H24B	0.99
C10—N4	1.476 (2)	C25—N7	1.455 (2)
C10—H10A	0.99	C25—N5	1.516 (2)
C10—H10B	0.99	C25—H25A	0.99
C11—N2	1.454 (2)	C25—H25B	0.99
C11—N1	1.513 (2)	C26—N6	1.471 (2)
C11—H11A	0.99	C26—N8	1.477 (2)
C11—H11B	0.99	C26—H26A	0.99
C12—N4	1.458 (2)	C26—H26B	0.99
C12—N1	1.513 (2)	B1—F1A	1.303 (11)
C12—H12A	0.99	B1—F3A	1.328 (8)
C12—H12B	0.99	B1—F4A	1.326 (16)
C13—N3	1.472 (2)	B1—F2B	1.357 (11)
C13—N2	1.474 (2)	B1—F3B	1.377 (10)
C13—H13A	0.99	B1—F2A	1.436 (7)
C13—H13B	0.99	B1—F4B	1.460 (17)
C14—C18	1.414 (3)	B1—F1B	1.483 (12)
C14—C15	1.430 (3)	B2—F6	1.352 (2)
C14—Fe2	2.1079 (18)	B2—F8	1.383 (2)
C14—H14	1	B2—F5	1.393 (3)
C15—C16	1.410 (3)	B2—F7	1.411 (2)
C15—Fe2	2.0838 (18)	N1—Fe1	2.0459 (15)
C15—H15	1	N5—Fe2	2.0490 (14)
			
C2—C1—C5	107.24 (19)	N5—C25—H25A	109.1
C2—C1—Fe1	70.29 (12)	N7—C25—H25B	109.1
C5—C1—Fe1	70.66 (12)	N5—C25—H25B	109.1
C2—C1—H1	126.4	H25A—C25—H25B	107.8
C5—C1—H1	126.4	N6—C26—N8	111.42 (14)
Fe1—C1—H1	126.4	N6—C26—H26A	109.3
C1—C2—C3	108.7 (2)	N8—C26—H26A	109.3
C1—C2—Fe1	70.15 (11)	N6—C26—H26B	109.3
C3—C2—Fe1	71.05 (11)	N8—C26—H26B	109.3
C1—C2—H2	125.6	H26A—C26—H26B	108
C3—C2—H2	125.6	F1A—B1—F3A	96 (2)
Fe1—C2—H2	125.6	F1A—B1—F4A	101.6 (9)
C4—C3—C2	107.1 (2)	F3A—B1—F4A	116.1 (7)
C4—C3—Fe1	71.38 (12)	F1A—B1—F2B	118.5 (8)
C2—C3—Fe1	69.24 (11)	F3A—B1—F2B	110.5 (10)
C4—C3—H3	126.4	F4A—B1—F2B	112.9 (11)
C2—C3—H3	126.4	F1A—B1—F3B	121.9 (14)
Fe1—C3—H3	126.4	F4A—B1—F3B	104.0 (8)
C5—C4—C3	109.10 (19)	F2B—B1—F3B	97.6 (10)
C5—C4—Fe1	69.50 (12)	F1A—B1—F2A	109.6 (8)
C3—C4—Fe1	69.81 (12)	F3A—B1—F2A	115.0 (6)
C5—C4—H4	125.4	F4A—B1—F2A	115.2 (10)
C3—C4—H4	125.4	F3B—B1—F2A	105.0 (7)
Fe1—C4—H4	125.4	F1A—B1—F4B	112.2 (9)
C4—C5—C1	107.8 (2)	F3A—B1—F4B	121.6 (11)
C4—C5—Fe1	71.71 (13)	F2B—B1—F4B	99.2 (10)
C1—C5—Fe1	69.35 (12)	F3B—B1—F4B	104.1 (10)
C4—C5—H5	126.1	F2A—B1—F4B	102.2 (8)
C1—C5—H5	126.1	F3A—B1—F1B	107.8 (14)
Fe1—C5—H5	126.1	F4A—B1—F1B	106.2 (9)
O1—C6—Fe1	173.88 (19)	F2B—B1—F1B	102.2 (7)
O2—C7—Fe1	175.25 (17)	F3B—B1—F1B	133.4 (8)
N3—C8—N1	112.50 (14)	F2A—B1—F1B	93.2 (6)
N3—C8—H8A	109.1	F4B—B1—F1B	113.5 (10)
N1—C8—H8A	109.1	F6—B2—F8	111.51 (17)
N3—C8—H8B	109.1	F6—B2—F5	112.30 (17)
N1—C8—H8B	109.1	F8—B2—F5	108.44 (17)
H8A—C8—H8B	107.8	F6—B2—F7	110.39 (15)
N3—C9—N4	111.56 (14)	F8—B2—F7	107.23 (17)
N3—C9—H9A	109.3	F5—B2—F7	106.73 (16)
N4—C9—H9A	109.3	C12—N1—C11	107.23 (13)
N3—C9—H9B	109.3	C12—N1—C8	106.19 (13)
N4—C9—H9B	109.3	C11—N1—C8	106.39 (13)
H9A—C9—H9B	108	C12—N1—Fe1	112.67 (10)
N2—C10—N4	111.52 (15)	C11—N1—Fe1	112.30 (10)
N2—C10—H10A	109.3	C8—N1—Fe1	111.63 (10)
N4—C10—H10A	109.3	C11—N2—C13	109.43 (15)
N2—C10—H10B	109.3	C11—N2—C10	108.47 (14)
N4—C10—H10B	109.3	C13—N2—C10	108.45 (15)
H10A—C10—H10B	108	C8—N3—C9	109.42 (14)
N2—C11—N1	112.18 (14)	C8—N3—C13	108.63 (15)
N2—C11—H11A	109.2	C9—N3—C13	108.34 (15)
N1—C11—H11A	109.2	C12—N4—C9	108.63 (15)
N2—C11—H11B	109.2	C12—N4—C10	108.82 (14)
N1—C11—H11B	109.2	C9—N4—C10	108.48 (14)
H11A—C11—H11B	107.9	C21—N5—C25	106.91 (13)
N4—C12—N1	112.32 (14)	C21—N5—C24	107.17 (13)
N4—C12—H12A	109.1	C25—N5—C24	105.93 (13)
N1—C12—H12A	109.1	C21—N5—Fe2	111.19 (10)
N4—C12—H12B	109.1	C25—N5—Fe2	111.84 (10)
N1—C12—H12B	109.1	C24—N5—Fe2	113.40 (10)
H12A—C12—H12B	107.9	C21—N6—C26	108.70 (14)
N3—C13—N2	111.41 (15)	C21—N6—C22	109.35 (14)
N3—C13—H13A	109.3	C26—N6—C22	108.65 (14)
N2—C13—H13A	109.3	C25—N7—C22	108.59 (14)
N3—C13—H13B	109.3	C25—N7—C23	109.26 (14)
N2—C13—H13B	109.3	C22—N7—C23	108.29 (14)
H13A—C13—H13B	108	C24—N8—C23	108.68 (14)
C18—C14—C15	107.45 (17)	C24—N8—C26	108.82 (14)
C18—C14—Fe2	71.86 (10)	C23—N8—C26	108.60 (14)
C15—C14—Fe2	69.15 (10)	C6—Fe1—C7	93.95 (9)
C18—C14—H14	126.3	C6—Fe1—N1	95.05 (8)
C15—C14—H14	126.3	C7—Fe1—N1	93.38 (7)
Fe2—C14—H14	126.3	C6—Fe1—C1	113.22 (9)
C16—C15—C14	108.27 (17)	C7—Fe1—C1	88.13 (8)
C16—C15—Fe2	70.30 (10)	N1—Fe1—C1	151.53 (7)
C14—C15—Fe2	70.96 (10)	C6—Fe1—C2	86.60 (9)
C16—C15—H15	125.9	C7—Fe1—C2	119.82 (8)
C14—C15—H15	125.9	N1—Fe1—C2	146.62 (7)
Fe2—C15—H15	125.9	C1—Fe1—C2	39.56 (8)
C15—C16—C17	107.98 (17)	C6—Fe1—C5	151.87 (9)
C15—C16—Fe2	70.17 (10)	C7—Fe1—C5	93.38 (9)
C17—C16—Fe2	71.26 (10)	N1—Fe1—C5	111.58 (7)
C15—C16—H16	126	C1—Fe1—C5	39.99 (8)
C17—C16—H16	126	C2—Fe1—C5	66.26 (9)
Fe2—C16—H16	126	C6—Fe1—C3	98.24 (10)
C18—C17—C16	107.63 (17)	C7—Fe1—C3	154.68 (8)
C18—C17—Fe2	71.57 (11)	N1—Fe1—C3	107.47 (7)
C16—C17—Fe2	68.98 (10)	C1—Fe1—C3	66.67 (8)
C18—C17—H17	126.2	C2—Fe1—C3	39.71 (8)
C16—C17—H17	126.2	C5—Fe1—C3	65.97 (9)
Fe2—C17—H17	126.2	C6—Fe1—C4	135.95 (10)
C14—C18—C17	108.64 (17)	C7—Fe1—C4	129.04 (9)
C14—C18—Fe2	69.27 (10)	N1—Fe1—C4	91.81 (7)
C17—C18—Fe2	69.57 (11)	C1—Fe1—C4	65.90 (8)
C14—C18—H18	125.7	C2—Fe1—C4	65.45 (8)
C17—C18—H18	125.7	C5—Fe1—C4	38.79 (9)
Fe2—C18—H18	125.7	C3—Fe1—C4	38.81 (9)
O3—C19—Fe2	174.69 (17)	C19—Fe2—C20	94.22 (8)
O4—C20—Fe2	174.77 (16)	C19—Fe2—N5	93.83 (7)
N6—C21—N5	111.92 (14)	C20—Fe2—N5	94.39 (7)
N6—C21—H21A	109.2	C19—Fe2—C15	86.20 (8)
N5—C21—H21A	109.2	C20—Fe2—C15	117.84 (8)
N6—C21—H21B	109.2	N5—Fe2—C15	147.71 (7)
N5—C21—H21B	109.2	C19—Fe2—C16	114.42 (8)
H21A—C21—H21B	107.9	C20—Fe2—C16	87.55 (8)
N7—C22—N6	111.47 (14)	N5—Fe2—C16	151.50 (7)
N7—C22—H22A	109.3	C15—Fe2—C16	39.53 (7)
N6—C22—H22A	109.3	C19—Fe2—C14	96.32 (8)
N7—C22—H22B	109.3	C20—Fe2—C14	154.12 (8)
N6—C22—H22B	109.3	N5—Fe2—C14	108.38 (6)
H22A—C22—H22B	108	C15—Fe2—C14	39.89 (7)
N7—C23—N8	111.49 (14)	C16—Fe2—C14	66.57 (7)
N7—C23—H23A	109.3	C19—Fe2—C17	152.07 (8)
N8—C23—H23A	109.3	C20—Fe2—C17	94.59 (8)
N7—C23—H23B	109.3	N5—Fe2—C17	111.83 (7)
N8—C23—H23B	109.3	C15—Fe2—C17	66.29 (8)
H23A—C23—H23B	108	C16—Fe2—C17	39.76 (8)
N8—C24—N5	112.23 (13)	C14—Fe2—C17	65.96 (7)
N8—C24—H24A	109.2	C19—Fe2—C18	133.73 (8)
N5—C24—H24A	109.2	C20—Fe2—C18	130.92 (8)
N8—C24—H24B	109.2	N5—Fe2—C18	92.38 (6)
N5—C24—H24B	109.2	C15—Fe2—C18	65.71 (7)
H24A—C24—H24B	107.9	C16—Fe2—C18	65.79 (7)
N7—C25—N5	112.53 (14)	C14—Fe2—C18	38.86 (7)
N7—C25—H25A	109.1	C17—Fe2—C18	38.86 (7)
			
C5—C1—C2—C3	−0.5 (2)	C3—C2—Fe1—C1	−119.0 (2)
Fe1—C1—C2—C3	60.88 (14)	C1—C2—Fe1—C5	38.58 (13)
C5—C1—C2—Fe1	−61.34 (13)	C3—C2—Fe1—C5	−80.39 (15)
C1—C2—C3—C4	1.4 (2)	C1—C2—Fe1—C3	119.0 (2)
Fe1—C2—C3—C4	61.71 (14)	C1—C2—Fe1—C4	81.25 (14)
C1—C2—C3—Fe1	−60.31 (14)	C3—C2—Fe1—C4	−37.72 (14)
C2—C3—C4—C5	−1.8 (2)	C4—C5—Fe1—C6	96.1 (2)
Fe1—C3—C4—C5	58.52 (14)	C1—C5—Fe1—C6	−21.8 (3)
C2—C3—C4—Fe1	−60.33 (14)	C4—C5—Fe1—C7	−159.01 (13)
C3—C4—C5—C1	1.5 (2)	C1—C5—Fe1—C7	83.04 (13)
Fe1—C4—C5—C1	60.24 (14)	C4—C5—Fe1—N1	−64.02 (13)
C3—C4—C5—Fe1	−58.71 (15)	C1—C5—Fe1—N1	178.02 (11)
C2—C1—C5—C4	−0.6 (2)	C4—C5—Fe1—C1	117.95 (18)
Fe1—C1—C5—C4	−61.75 (14)	C4—C5—Fe1—C2	79.79 (13)
C2—C1—C5—Fe1	61.10 (14)	C1—C5—Fe1—C2	−38.17 (12)
C18—C14—C15—C16	1.3 (2)	C4—C5—Fe1—C3	36.18 (12)
Fe2—C14—C15—C16	−60.70 (13)	C1—C5—Fe1—C3	−81.77 (13)
C18—C14—C15—Fe2	62.03 (12)	C1—C5—Fe1—C4	−117.95 (18)
C14—C15—C16—C17	−0.5 (2)	C4—C3—Fe1—C6	168.18 (14)
Fe2—C15—C16—C17	−61.57 (13)	C2—C3—Fe1—C6	−74.45 (15)
C14—C15—C16—Fe2	61.11 (13)	C4—C3—Fe1—C7	−73.9 (2)
C15—C16—C17—C18	−0.6 (2)	C2—C3—Fe1—C7	43.5 (3)
Fe2—C16—C17—C18	−61.48 (13)	C4—C3—Fe1—N1	70.21 (14)
C15—C16—C17—Fe2	60.88 (13)	C2—C3—Fe1—N1	−172.42 (13)
C15—C14—C18—C17	−1.7 (2)	C4—C3—Fe1—C1	−80.01 (14)
Fe2—C14—C18—C17	58.57 (13)	C2—C3—Fe1—C1	37.36 (14)
C15—C14—C18—Fe2	−60.29 (12)	C4—C3—Fe1—C2	−117.4 (2)
C16—C17—C18—C14	1.4 (2)	C4—C3—Fe1—C5	−36.17 (13)
Fe2—C17—C18—C14	−58.39 (13)	C2—C3—Fe1—C5	81.21 (15)
C16—C17—C18—Fe2	59.83 (13)	C2—C3—Fe1—C4	117.4 (2)
N4—C12—N1—C11	56.03 (18)	C5—C4—Fe1—C6	−137.60 (15)
N4—C12—N1—C8	−57.41 (18)	C3—C4—Fe1—C6	−16.95 (19)
N4—C12—N1—Fe1	−179.91 (11)	C5—C4—Fe1—C7	27.41 (17)
N2—C11—N1—C12	−56.77 (18)	C3—C4—Fe1—C7	148.06 (13)
N2—C11—N1—C8	56.53 (18)	C5—C4—Fe1—N1	123.24 (13)
N2—C11—N1—Fe1	178.94 (11)	C3—C4—Fe1—N1	−116.11 (13)
N3—C8—N1—C12	56.63 (18)	C5—C4—Fe1—C1	−38.46 (13)
N3—C8—N1—C11	−57.39 (18)	C3—C4—Fe1—C1	82.19 (14)
N3—C8—N1—Fe1	179.78 (12)	C5—C4—Fe1—C2	−82.06 (14)
N1—C11—N2—C13	−58.67 (19)	C3—C4—Fe1—C2	38.59 (13)
N1—C11—N2—C10	59.46 (19)	C3—C4—Fe1—C5	120.65 (18)
N3—C13—N2—C11	59.3 (2)	C5—C4—Fe1—C3	−120.65 (18)
N3—C13—N2—C10	−58.9 (2)	C21—N5—Fe2—C19	−66.78 (12)
N4—C10—N2—C11	−60.45 (19)	C25—N5—Fe2—C19	52.66 (12)
N4—C10—N2—C13	58.31 (19)	C24—N5—Fe2—C19	172.37 (12)
N1—C8—N3—C9	−58.50 (19)	C21—N5—Fe2—C20	−161.31 (12)
N1—C8—N3—C13	59.60 (19)	C25—N5—Fe2—C20	−41.87 (12)
N4—C9—N3—C8	58.99 (19)	C24—N5—Fe2—C20	77.84 (12)
N4—C9—N3—C13	−59.29 (19)	C21—N5—Fe2—C15	22.16 (18)
N2—C13—N3—C8	−59.4 (2)	C25—N5—Fe2—C15	141.60 (14)
N2—C13—N3—C9	59.4 (2)	C24—N5—Fe2—C15	−98.69 (15)
N1—C12—N4—C9	59.75 (18)	C21—N5—Fe2—C16	105.75 (16)
N1—C12—N4—C10	−58.17 (19)	C25—N5—Fe2—C16	−134.81 (15)
N3—C9—N4—C12	−59.39 (19)	C24—N5—Fe2—C16	−15.1 (2)
N3—C9—N4—C10	58.75 (19)	C21—N5—Fe2—C14	31.19 (13)
N2—C10—N4—C12	59.85 (19)	C25—N5—Fe2—C14	150.62 (11)
N2—C10—N4—C9	−58.16 (19)	C24—N5—Fe2—C14	−89.67 (12)
N6—C21—N5—C25	56.37 (18)	C21—N5—Fe2—C17	101.96 (12)
N6—C21—N5—C24	−56.85 (17)	C25—N5—Fe2—C17	−138.60 (11)
N6—C21—N5—Fe2	178.70 (11)	C24—N5—Fe2—C17	−18.89 (13)
N7—C25—N5—C21	−56.97 (18)	C21—N5—Fe2—C18	67.34 (12)
N7—C25—N5—C24	57.09 (17)	C25—N5—Fe2—C18	−173.22 (12)
N7—C25—N5—Fe2	−178.90 (11)	C24—N5—Fe2—C18	−53.51 (12)
N8—C24—N5—C21	56.10 (18)	C16—C15—Fe2—C19	−137.02 (13)
N8—C24—N5—C25	−57.77 (17)	C14—C15—Fe2—C19	104.57 (12)
N8—C24—N5—Fe2	179.20 (11)	C16—C15—Fe2—C20	−44.25 (14)
N5—C21—N6—C26	59.81 (18)	C14—C15—Fe2—C20	−162.66 (11)
N5—C21—N6—C22	−58.66 (18)	C16—C15—Fe2—N5	131.83 (13)
N8—C26—N6—C21	−60.71 (18)	C14—C15—Fe2—N5	13.42 (19)
N8—C26—N6—C22	58.20 (18)	C14—C15—Fe2—C16	−118.41 (17)
N7—C22—N6—C21	59.69 (19)	C16—C15—Fe2—C14	118.41 (17)
N7—C22—N6—C26	−58.81 (19)	C16—C15—Fe2—C17	38.09 (12)
N5—C25—N7—C22	59.10 (19)	C14—C15—Fe2—C17	−80.32 (12)
N5—C25—N7—C23	−58.82 (18)	C16—C15—Fe2—C18	80.80 (12)
N6—C22—N7—C25	−59.46 (19)	C14—C15—Fe2—C18	−37.61 (11)
N6—C22—N7—C23	59.07 (18)	C15—C16—Fe2—C19	48.34 (14)
N8—C23—N7—C25	58.91 (19)	C17—C16—Fe2—C19	166.30 (11)
N8—C23—N7—C22	−59.20 (18)	C15—C16—Fe2—C20	141.86 (12)
N5—C24—N8—C23	59.99 (18)	C17—C16—Fe2—C20	−100.18 (12)
N5—C24—N8—C26	−58.10 (18)	C15—C16—Fe2—N5	−123.47 (15)
N7—C23—N8—C24	−59.41 (18)	C17—C16—Fe2—N5	−5.5 (2)
N7—C23—N8—C26	58.82 (18)	C17—C16—Fe2—C15	117.96 (16)
N6—C26—N8—C24	59.87 (18)	C15—C16—Fe2—C14	−37.94 (11)
N6—C26—N8—C23	−58.28 (19)	C17—C16—Fe2—C14	80.02 (12)
C12—N1—Fe1—C6	166.15 (12)	C15—C16—Fe2—C17	−117.96 (16)
C11—N1—Fe1—C6	−72.64 (13)	C15—C16—Fe2—C18	−80.58 (12)
C8—N1—Fe1—C6	46.76 (13)	C17—C16—Fe2—C18	37.38 (11)
C12—N1—Fe1—C7	71.87 (12)	C18—C14—Fe2—C19	166.14 (12)
C11—N1—Fe1—C7	−166.91 (12)	C15—C14—Fe2—C19	−76.31 (12)
C8—N1—Fe1—C7	−47.52 (12)	C18—C14—Fe2—C20	−80.4 (2)
C12—N1—Fe1—C1	−20.4 (2)	C15—C14—Fe2—C20	37.1 (2)
C11—N1—Fe1—C1	100.77 (17)	C18—C14—Fe2—N5	69.96 (12)
C8—N1—Fe1—C1	−139.84 (15)	C15—C14—Fe2—N5	−172.49 (11)
C12—N1—Fe1—C2	−102.37 (16)	C18—C14—Fe2—C15	−117.55 (16)
C11—N1—Fe1—C2	18.8 (2)	C18—C14—Fe2—C16	−79.95 (12)
C8—N1—Fe1—C2	138.24 (15)	C15—C14—Fe2—C16	37.59 (11)
C12—N1—Fe1—C5	−23.11 (14)	C18—C14—Fe2—C17	−36.35 (11)
C11—N1—Fe1—C5	98.10 (13)	C15—C14—Fe2—C17	81.20 (12)
C8—N1—Fe1—C5	−142.50 (12)	C15—C14—Fe2—C18	117.55 (16)
C12—N1—Fe1—C3	−93.56 (13)	C18—C17—Fe2—C19	90.6 (2)
C11—N1—Fe1—C3	27.65 (14)	C16—C17—Fe2—C19	−27.4 (2)
C8—N1—Fe1—C3	147.05 (12)	C18—C17—Fe2—C20	−161.38 (12)
C12—N1—Fe1—C4	−57.41 (13)	C16—C17—Fe2—C20	80.58 (12)
C11—N1—Fe1—C4	63.80 (13)	C18—C17—Fe2—N5	−64.79 (12)
C8—N1—Fe1—C4	−176.80 (13)	C16—C17—Fe2—N5	177.17 (10)
C2—C1—Fe1—C6	51.64 (15)	C18—C17—Fe2—C15	80.16 (12)
C5—C1—Fe1—C6	168.99 (13)	C16—C17—Fe2—C15	−37.88 (11)
C2—C1—Fe1—C7	145.14 (13)	C18—C17—Fe2—C16	118.04 (16)
C5—C1—Fe1—C7	−97.51 (13)	C18—C17—Fe2—C14	36.36 (11)
C2—C1—Fe1—N1	−121.21 (16)	C16—C17—Fe2—C14	−81.68 (12)
C5—C1—Fe1—N1	−3.9 (2)	C16—C17—Fe2—C18	−118.04 (16)
C5—C1—Fe1—C2	117.35 (18)	C14—C18—Fe2—C19	−19.24 (16)
C2—C1—Fe1—C5	−117.35 (18)	C17—C18—Fe2—C19	−139.60 (13)
C2—C1—Fe1—C3	−37.49 (13)	C14—C18—Fe2—C20	145.27 (12)
C5—C1—Fe1—C3	79.86 (14)	C17—C18—Fe2—C20	24.91 (15)
C2—C1—Fe1—C4	−80.03 (14)	C14—C18—Fe2—N5	−116.84 (11)
C5—C1—Fe1—C4	37.32 (13)	C17—C18—Fe2—N5	122.80 (11)
C1—C2—Fe1—C6	−133.79 (14)	C14—C18—Fe2—C15	38.60 (11)
C3—C2—Fe1—C6	107.23 (15)	C17—C18—Fe2—C15	−81.77 (12)
C1—C2—Fe1—C7	−41.18 (16)	C14—C18—Fe2—C16	82.13 (12)
C3—C2—Fe1—C7	−160.16 (14)	C17—C18—Fe2—C16	−38.23 (11)
C1—C2—Fe1—N1	132.19 (14)	C17—C18—Fe2—C14	−120.37 (16)
C3—C2—Fe1—N1	13.2 (2)	C14—C18—Fe2—C17	120.37 (16)
